# Supplementary material for: Structural Features and Immunomodulatory Effects of Water-Extractable Polysaccharides from Macrolepiota procera (Scop.) Singer
Source: J Fungi (Basel). 2022 Aug 13;8(8):848. doi: 10.3390/jof8080848 (PMC9410249; doi:10.3390/jof8080848)
Supplement: Supplementary file 1 [file jof-08-00848-s001.zip › Supplementary materials_Final.pdf]

Journal of *Fungi*

MDPI

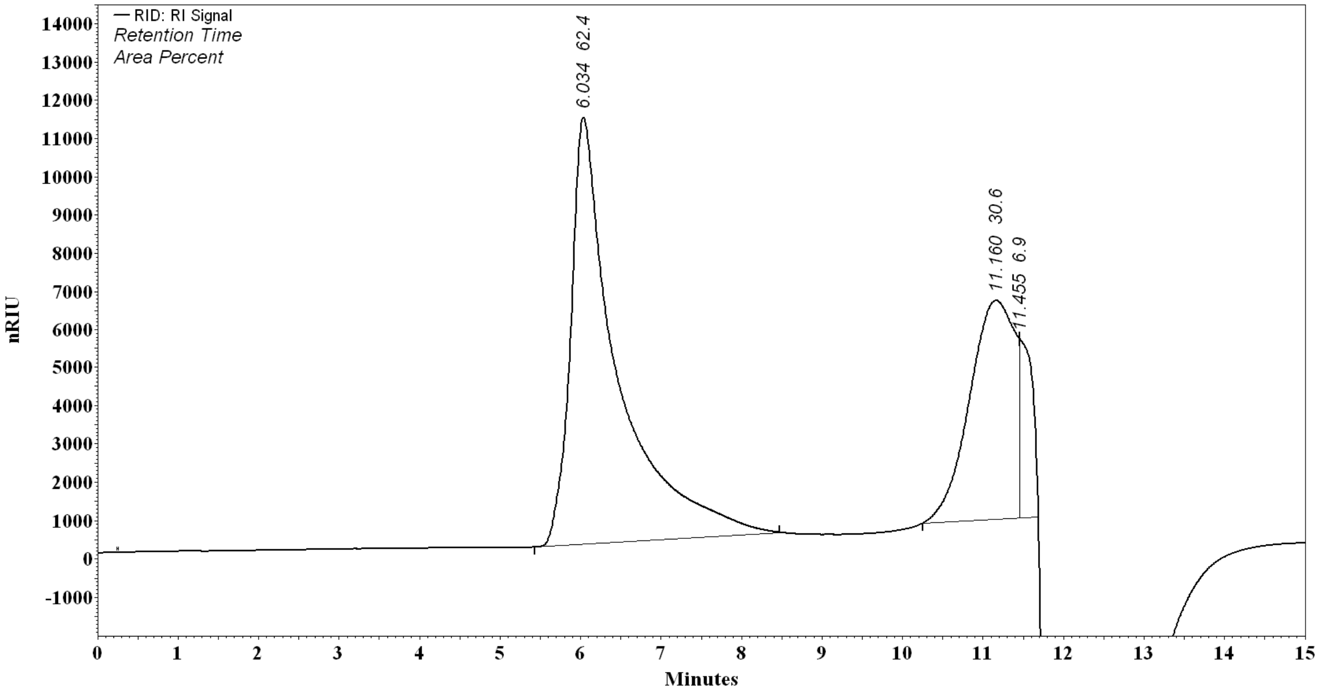

Figure S1. Molecular weight distribution of the water-extractable polysaccharide complex from the fruiting bodies of *M. procera* (MP-PSC)

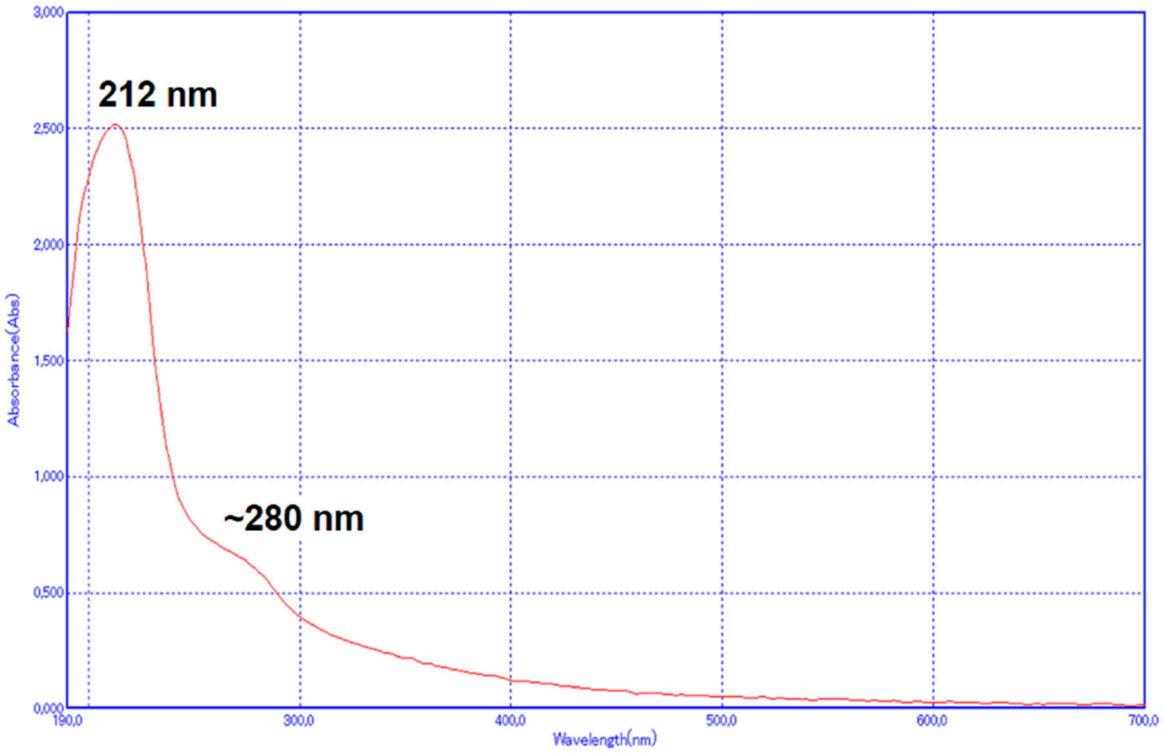

Figure S2. UV-Vis spectrum (1 mg/mL) of MP-PSC.

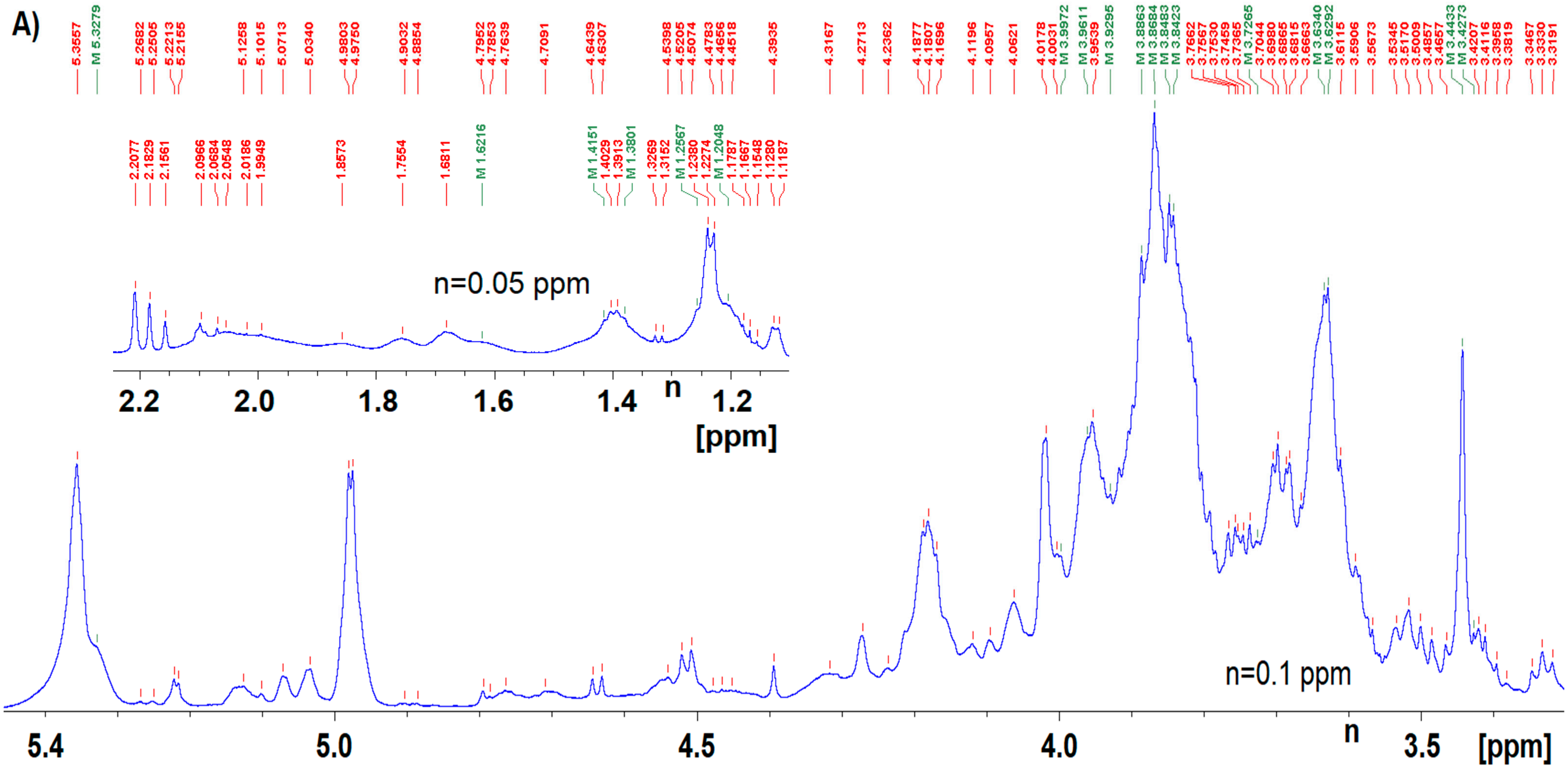
A)
5.3557
M 5.3279
5.2682
5.2505
5.2213
5.2155
5.1258
5.1015
5.0713
5.0340
4.9803
4.9750
4.9032
4.8854
4.7952
4.7853
4.7639
4.7091
4.6439
4.6307
4.5398
4.5205
4.5074
4.4783
4.4656
4.4518
4.3935
4.3167
4.2713
4.2362
4.1877
4.1807
4.1696
4.1196
4.0957
4.0621
4.0178
4.0031
M 3.9972
M 3.9611
3.9539
M 3.9295
M 3.8863
M 3.8684
M 3.8483
M 3.8423
3.7662
3.7567
3.7530
3.7459
3.7365
M 3.7265
3.7044
3.6980
3.6865
3.6815
3.6663
M 3.6340
M 3.6292
3.6115
3.5906
3.5673
3.5345
3.5170
3.5009
3.4857
3.4657
M 3.4433
M 3.4273
3.4207
3.4116
3.3958
3.3819
3.3467
3.3330
3.3191
2.2077
2.1829
2.1561
2.0966
2.0684
2.0548
2.0186
1.9949
1.8573
1.7554
1.6811
M 1.6216
M 1.4151
1.4029
1.3913
M 1.3801
1.3269
1.3152
M 1.2567
1.2380
1.2274
M 1.2048
1.1787
1.1667
1.1548
1.1280
1.1187
n=0.05 ppm
2.2
2.0
1.8
1.6
1.4
n
1.2
[ppm]
n=0.1 ppm
5.4
5.0
4.5
4.0
n
3.5
[ppm]

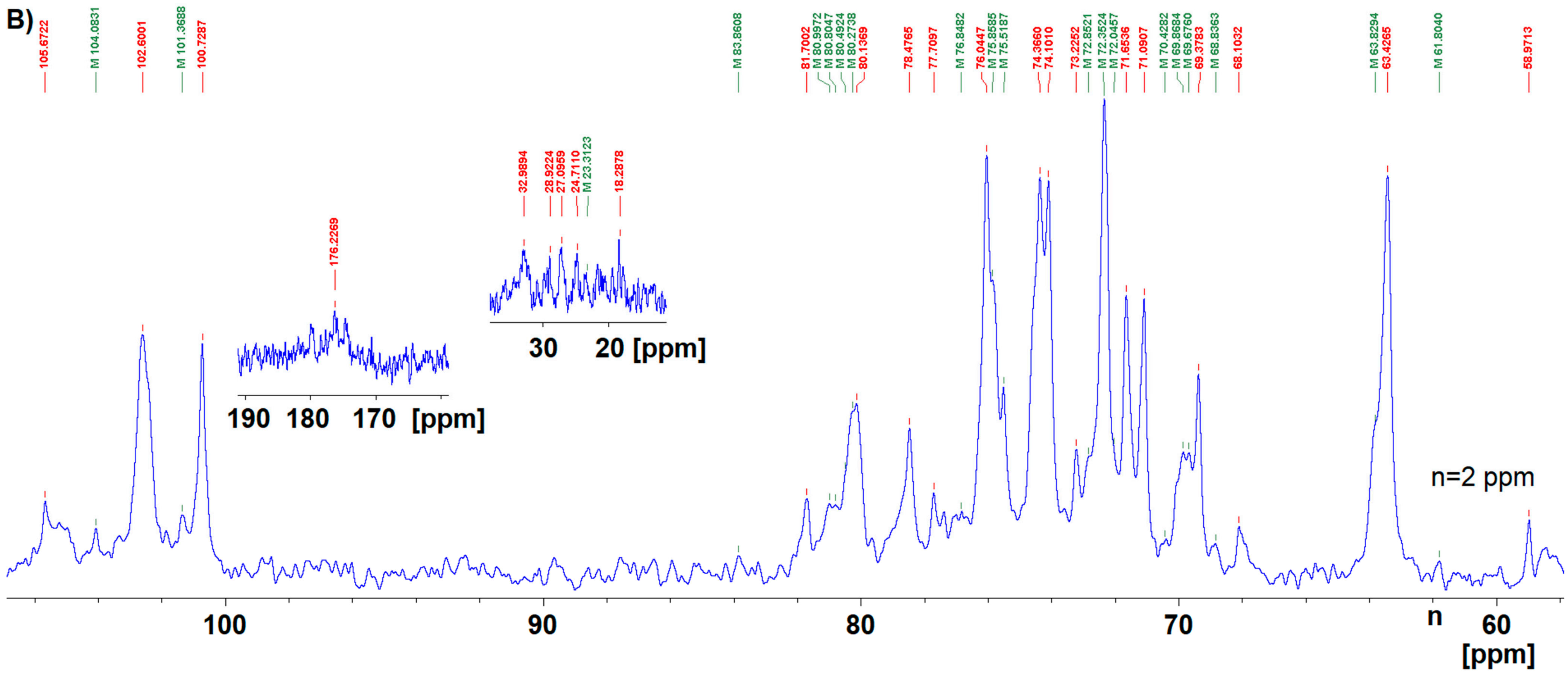

Figure S3. Fragments of ^{1}H spectrum (**A)**) and ^{13}C spectrum (**B)**) of MP-PSC. Sodium 4,4-dimethyl-4-silapentane-sulfonate was used as an internal standard.

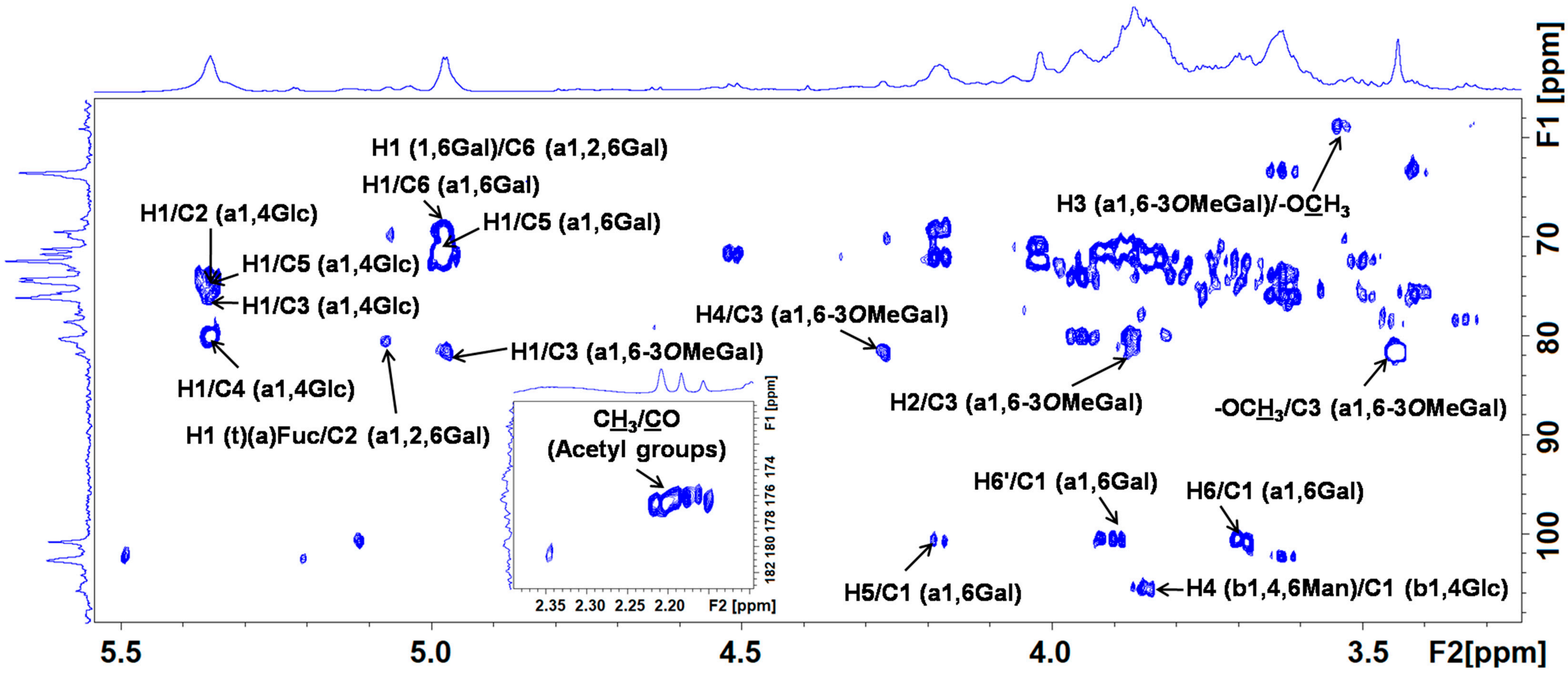

Figure S4. Fragments of $^1H/^{13}C$ HMBC spectrum of MP-PSC. Sodium 4,4-dimethyl-4-silapentane-sulfonate was used as an internal standard. Annotation: intra- and inter-residue $^1H/^{13}C$ correlations are shown, as α (a) and β (b) anomeric configurations, and glycosidic linkage types are added in brackets. Abbreviations: Me (methyl group), t (terminal).

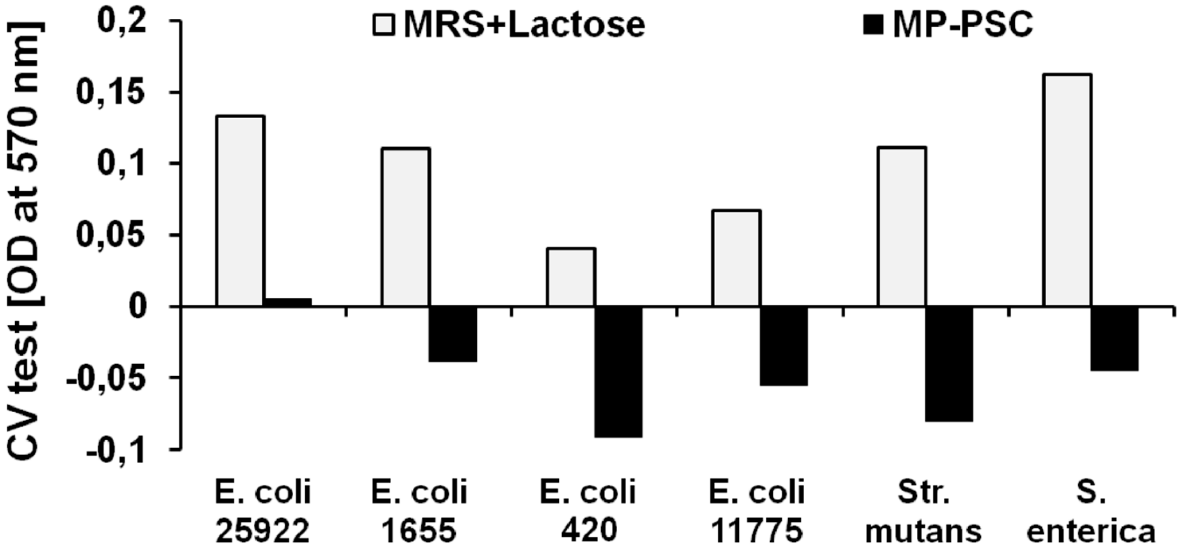

Figure S5. *In vitro* evaluation of biofilm formation from clinical and reference pathogenic strains, after 96 h of incubation at 37°C in a modified MRS broth with added MP-PSC (2%, w/v), as a sole carbon source. Lactose (modified MRS broth + Lactose, 2%, w/v) served as a control. Biofilm formation was determined by the crystal violet (CV) test.
